# The application of Industry 4.0 technologies in sustainable logistics: a systematic literature review (2012–2020) to explore future research opportunities

**DOI:** 10.1007/s11356-021-17693-y

**Published:** 2021-12-10

**Authors:** Xu Sun, Hao Yu, Wei Deng Solvang, Yi Wang, Kesheng Wang

**Affiliations:** 1 Department of Industrial Engineering, UiT—The Arctic University of Norway, Narvik, Norway; 2 School of Business, University of Plymouth, Plymouth, Devon UK; 3 Department of Mechanical and Industrial Engineering, Norwegian University of Science and Technology, Trondheim, Norway

**Keywords:** Sustainable logistics, Green logistics, Industry 4.0, Smart technology, Literature review, Bibliometric analysis

## Abstract

Nowadays, the market competition becomes increasingly fierce due to diversified customer needs, stringent environmental requirements, and global competitors. One of the most important factors for companies to not only survive but also thrive in today’s competitive market is their logistics performance. This paper aims, through a systematic literature analysis of 115 papers from 2012 to 2020, at presenting quantitative insights and comprehensive overviews of the current and future research landscapes of sustainable logistics in the Industry 4.0 era. The results show that Industry 4.0 technologies provide opportunities for improving the economic efficiency, environmental performance, and social impact of logistics sectors. However, several challenges arise with this technological transformation, i.e., trade-offs among different sustainability indicators, unclear benefits, lifecycle environmental impact, inequity issues, and technology maturity. Thus, to better tackle the current research gaps, future suggestions are given to focus on the balance among different sustainability indicators through the entire lifecycle, human-centric technological transformation, system integration and digital twin, semi-autonomous transportation solutions, smart reverse logistics, and so forth.

## Introduction 

With the increasing concerns on environmental pollution, resource depletion, and climate change from the whole society, enterprises must transform their businesses and operations into more sustainable ways (Rauter et al. 2017). Recent studies have shown that more focus and investments on enterprises’ sustainable practices not only help them to build up a socially responsible image but also improve their overall sustainable performance in economic and environmental dimensions (Allaoui et al. 2019). Logistics links different operations and players within a supply chain and is a vital part that largely determines a company’s overall effectiveness and resource efficiency (Qaiser et al. 2017). Managing a logistics system involves several related activities, i.e., warehousing, inventory handling, information services, and transportation, and any decisions may influence a large number of stakeholders in either positive or negative ways (Murphy and Poist 2003). The effectiveness and sustainability of a logistics system determine the long-term competitiveness and the success of an enterprise. Therefore, new methods are investigated by both academia and industrial practitioners to improve the economic, environmental, and social sustainability of logistics activities.

The recent technological advancement and innovation of Industry 4.0 have provided new opportunities for enterprises to achieve value creation and proposition through satisfying individualized customer demands responsively and cost-effectively (Wang et al. 2017). This has not only led to a shift of the manufacturing paradigm but also drastically affected the way of logistics operations toward a high level of digitalization, connectivity, intelligence, integration, and responsiveness (Winkelhaus and Grosse 2020). Even though Industry 4.0 provides new opportunities for enterprises to enhance their sustainable logistics practices, the operational transformation by adopting these new technologies has, however, never been a painless endeavor, which may also encounter structural resistance at both intra- and inter-enterprise levels (Sony and Naik 2019). Thus, a systematic literature analysis is important to provide useful implications into the advantages and challenges of adopting new technologies in sustainable logistics, which can help with a successful transformation of a company in the coming digital era.

Previous literature reviews have provided comprehensive insights into sustainable logistics planning (Brandenburg et al. 2014; Qaiser et al. 2017), green and sustainable logistics practices (Ren et al. 2020; Dey et al. 2011; Martins et al. 2020), sustainable freight transport (Nenni et al. 2019; Álvarez and de la Calle 2011), and knowledge management in sustainable logistics (Evangelista and Durst 2015). To improve the intelligence, agility, and efficiency of logistics activities, recent studies have put predominant emphasis on the adoption of new technologies, e.g., big data analytics (Chalmeta and Santos-deLeón 2020), blockchain (Reddy et al. 2021), artificial intelligence (AI) (Riahi et al. 2021; Tirkolaee et al. 2021), internet of things (IoT) (Tijan et al. 2019), and additive manufacturing (AM) (Khorram Niaki and Nonino 2017). This trend has led to the new architecture of Logistics 4.0 (Wang 2016). Besides, several recent reviews have discussed the connection between Industry 4.0 and general sustainable practices (Roblek et al. 2020).

Table 1 shows the comparison of recent literature reviews related to Industry 4.0, sustainability, and logistics. As shown, the research focus has been predominantly given to the general sustainability and supply chain issues related to Industry 4.0. However, there is still a lack of systematic analyses focusing on linking sustainable logistics practices with different Industry 4.0 technologies. Logistics is traditionally a labor-intensive industry, which experiences significant changes in this digital transformation, and both positive and negative impacts on the economic, environmental, and social sustainability need thus to be better understood. Besides, the use of both bibliometric analysis and content analysis has not been fully exploited. Bibliometric analysis is a quantitative method that shows the network data visualization of the inter-connections of different literature in several dimensions, but it has been rarely used in the literature reviews of Industry 4.0 and sustainability, particularly in combination with content analysis.

**Table 1:** Relevant literature reviews related to Industry 4.0, sustainability, and logistics

Papers	Research method		Sample selection		Research focus and perspectives	Keywords			
	Bibliometric analysis	Content analysis	Horizon	Sample size		Industry 4.0	Sustainability	Logistics	Supply chain
Davarzani et al. (2016)	√		1975–2014	338	Green and sustainable maritime logistics		√	√	
Bag et al. (2018)		√	1998–2017	53	Industry 4.0 enablers of supply chain sustainability	√	√		√
Ranieri et al. (2018)		√	2012–2016	24	Innovative last-mile delivery systems			√	
Kazemi et al. (2019)	√	√	2000–2017	94	Reverse logistics and closed-loop supply chain			√	√
Nenni et al. (2019)		√	1997–2018	93	Sustainability of urban freight transport		√		
Tijan et al. (2019)		√	Until 2018	–	Blockchain technology in logistics			√	
Manavalan and Jayakrishna (2019)		√	2009–2018	–	IoT embedded sustainable supply chain	√			√
Martins et al. (2020)		√	Until 2019	45	Sustainable logistics considering TBL		√	√	
Ren et al. (2020)	√	√	1999–2019	306	Green and sustainable logistics		√	√	√
Chalmeta and Santos-deLeón (2020)	√		2009–2019	87	Industry 4.0 and big data in sustainable supply chain practices	√	√		√
Winkelhaus and Grosse (2020)		√	2005–2018	114	Industry 4.0 and logistics	√		√	
Roblek et al. (2020)		√	2010–2020	173	Industry 4.0 and sustainability	√	√		
Ejsmont et al. (2020)	√		2011–2020	162	Sustainability and Industry 4.0	√	√		
Ghobakhloo (2020)		√	2012–2019	72	Industry 4.0 and sustainability	√	√		
Furstenau et al. (2020)	√		2010–2019	894	Industry 4.0 and sustainability	√	√		
Birkel and Müller (2020)		√	2011–2019	55	Industry 4.0 for sustainable supply chain management	√	√		√
Margherita and Braccini (2020)		√	2009–2019	18	Industry 4.0 organizational impacts on sustainability	√	√		
Beier et al. (2020)		√	2013–2021	51	Industry 4.0 and socio-technical sustainability	√	√		
Grzybowska and Awasthi (2020)	√		1991–2018	892	Sustainable production and logistics		√	√	
Abdirad and Krishnan (2020)		√	2014–2018	56	Industry 4.0 in supply chain management	√		√	√
Jahani et al. (2021)		√	2015–2020	70	Industry 4.0 in the procurement processes of supply chains	√	√		√
Beltrami et al. (2021)		√	2011–2020	117	Industry 4.0 and sustainability	√	√		
This paper	√	√	2011–2020	115	Sustainable logistics enabled by Industry 4.0	√	√	√	

**Fig. 1:**
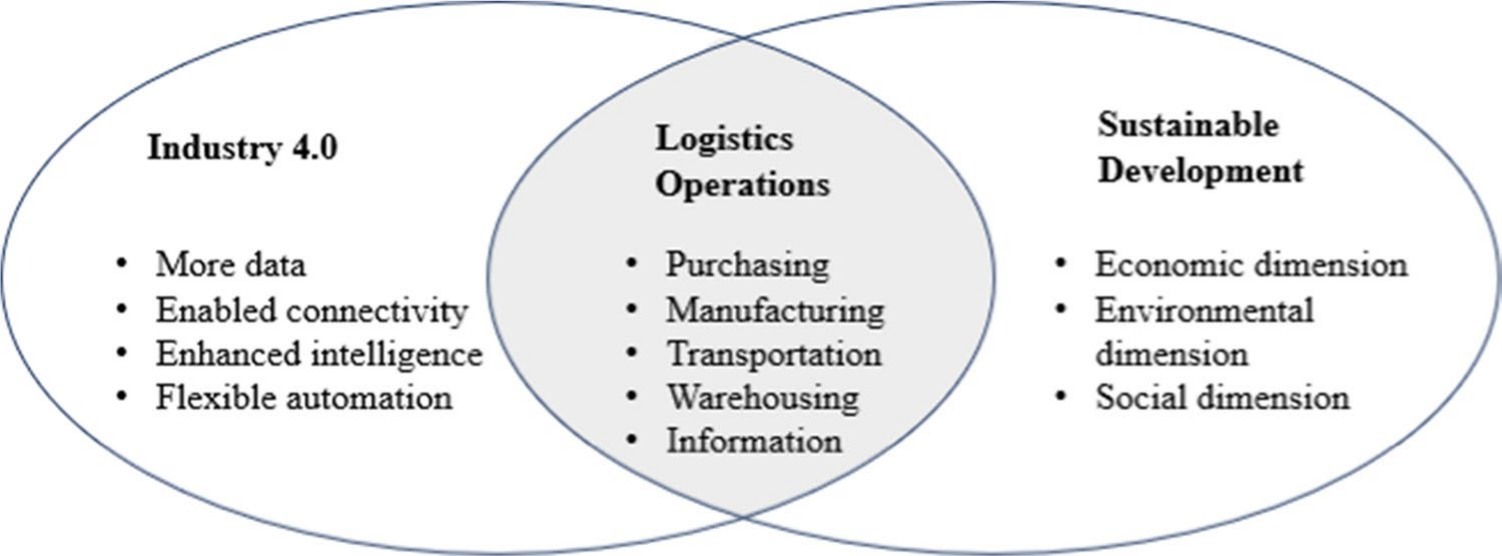
Research focus of this paper

Therefore, as shown in Fig. 1, this paper aims at filling the literature gap by conducting a systematic literature review to illustrate the current and future research landscapes of sustainable logistics in the Industry 4.0 era. The contributions are summarized as follows:

1. Using both bibliometric analysis and content analysis, we thoroughly explore the current research landscape that links sustainable logistics practices with various Industry 4.0 technologies.
2. We analyze both opportunities and challenges of adopting Industry 4.0 technologies in logistics sectors related to economic, environmental, and social sustainability.
3. We suggest nine future research directions to fill the current research gaps.
4. From the practical perspective, the discussions provide some successful examples of Industry 4.0 enabled transformation of logistics systems.

Following the introduction, the “Theoretical background” section gives the theoretical background of sustainable logistics and Industry 4.0. The “Research method” section presents the research method. The “Bibliometric analysis” and “Content analysis” sections provide the bibliometric analysis and content analysis. The opportunities, challenges, and future research suggestions are discussed in the “Discussions” section. Finally, the “Conclusions” section concludes the paper.

## Theoretical background

### Sustainable logistics

The word *logistics* appeared more than a century ago and was originally associated with the movement of troops and military supplies (Stevenson 2010). Over time, this word has been widely used to broadly describe the movement of physical goods among different locations (Lummus et al. 2001). Logistics deals with the entire cycle including pre-production, in-production, and post-production activities (Cavinato 1982). To fulfill customer needs at a satisfactory level, logistics aims at implementing a set of decisions including the purchase of raw materials, parts, and components, the handling and storage of inventories, and the transportation of goods from one location to another. The effectiveness and efficiency of the logistics system largely determine an enterprise’s performance in cost, customer satisfaction, and profitability. Recently, a concept called supply chain management has been used interchangeably to depict several logistics activities, but the scopes of the two words are not overlapped with each other (Larson and Halldorsson 2004). Several researchers suggest that supply chain management focuses more on forming strategies to manage the relationships and coordination among different partners (Christopher 2016, Winkelhaus and Grosse 2020), while logistics, on the other hand, emphasizes the implementation of these strategies to connect different companies with physical flows (Lummus et al. 2001). In this regard, logistics can be considered a subset of supply chain management (Winkelhaus and Grosse 2020), which focuses on the physical movement of goods and the relevant information flow.

Sustainable development has been focused on due to the concerns of increasingly severe environmental and social challenges. The widely accepted definition of sustainable development is “*to meet the needs of the present without compromising the ability of future generations to meet their own needs*” (Brundtland, 1987). Sustainable development is driven by three dimensions, namely, economic prosperity, environmental friendliness, and social fairness and equity, which are also known as the triple bottom line. The objective of a sustainable development society is to achieve harmony among these three dimensions. For tackling the global challenges related to hunger and poverty, health and well-being, environmental pollution, climate change, and global warming, the United Nations (UN) has recently set up 17 sustainable development goals, which are the call for actions to achieve a better future for all human beings by 2030 (UN 2015).

A drastic increase of companies has started to incorporate sustainability into logistics operations to enhance their social image and competitive advantage (Luthra and Mangla 2018). Sustainable logistics was initially focused on from the environmental perspective of lowering the ecological footprint related to logistics activities (Robert et al. 2005). The concept of green logistics was first proposed to reduce environmental impacts, e.g., GHG emissions (Dekker et al. 2012), energy consumption (Marchi and Zanoni 2017), etc., through better strategic designs and operational planning. Reverse logistics and closed-loop supply chain (CLSC) have been increasingly focused to achieve sustainable value re-creation from end-of-life (EOL) products (Solvang et al. 2007) and minimizing the environmental pollution from waste management (Gupta 2013, Govindan et al. 2015). However, improper disposal activities lead to risk exposure to both humans and the environment (Yu et al. 2020; Yu and Solvang 2016). Thus, recent research efforts have been given to minimize the ecological footprint of both forward and reverse logistics (Yu and Solvang 2020). Furthermore, not only the economic and environmental dimensions but also the social sustainability indicators, i.e., job creation and working environments, have been holistically considered in sustainable logistics. Therefore, sustainable logistics aims at balancing the socio-economic performance of a logistics system with its eco-environmental robustness in managing system activities. This balance embodies in making decisions by considering the interplay of different logistics functions, i.e., network configuration, transportation, purchasing, demand allocation, and resource management. The optimization of a sustainable logistics system is highly dependent on the ability to balance the trade-offs among the three dimensions of sustainability.

### Industry 4.0

Industry 4.0, or the fourth industrial revolution, was put forward at the Hannover Fair of Industrial Technologies in 2011 to enhance the competitiveness of the German manufacturing industry (Rojko 2017). At the global level, several countries have also launched their strategies, e.g., US *National Network for Manufacturing Innovation*, Japan’s *New Robot Strategy*, and China’s *Made in China 2025*, to strengthen their manufacturing industries by taking advantage of technological innovations (Lasi et al. 2014). While the past three industrial revolutions in history were the major results of mechanization, mass-production, and automated production (Rojko 2017), Industry 4.0 puts predominant focus on combining Internet-based communication technologies, digitalization, and future-oriented intelligent manufacturing technologies to build smart machines and systems, implement smart processes, and provide smart products and services (Lasi et al. 2014). Empowered by Industry 4.0 technologies, a smart production network can achieve real-time monitoring, responsive communications, autonomous operations, and smooth material flows. Technological advancement has provided opportunities and new business models for value creation and proposition from individualized customizations and service innovations (Esmaeilian et al. 2020). Based on previous studies (Sutawijaya and Nawangsari 2020; Strandhagen et al. 2017; Barreto et al. 2017), the 12 most important Industry 4.0 technologies are introduced as follows:

- *Internet of things (IoT)*: IoT refers to the network interconnection that possibly connects millions of physical objects with the Internet (Xia et al. 2012). It allows different smart devices can be interconnected, monitored, communicated, and controlled based on standard communication protocols to facilitate the transition of goods, services, and information (Barreto et al. 2017).
- *Cyber-physical system (CPS)*: CPS is the system integration of computational intelligence and physical elements, which enables effective interactions between the system and humans (Baheti and Gill 2011). CPS aims at achieving a high level of connectivity, intelligence, and automation by integrating both cyber and physical components (Zhang 2018). Thus, the level of CPS largely determines the successful implementation of Industry 4.0 (Qin et al. 2016).
- *Big data analytics*: Big data analytics is the state-of-the-art analytical capability to process a large volume of dynamic data with high velocity, high complexity, and high variety. The strategies and operations of a company or a system can be continuously evaluated through massive data analytics to obtain critical insights for better business planning and decision making (Wang et al. 2016).
- *Artificial intelligence (AI)*: AI refers to the computer systems and applications that perform tasks needing human intelligence (Pesapane et al. 2018), and it also has the capacity of learning and improving the thinking, perception, and action through training from data and algorithms (Helm et al. 2020). AI algorithms are widely used in many areas, e.g., routing, traffic management, maintenance, and security (Matlou and Abu-Mahfouz 2017).
- *Cloud technologies*: Cloud technologies provide a central platform for the storage and integration of configurable information technology (IT) resources, which enable the accessibility of data and resources from decentralized locations. Cloud technologies form the service-oriented architecture that links the concepts of Platform-as-a-Service (PaaS), Software-as-a-Service (SaaS), and Information-as-a-Service (IaaS) (Benotmane et al. 2017).
- *Blockchain:* Blockchain is an innovative way for implementing distributed ledger technologies that can be programmed to record and track any data by anyone without a central authority, and it is a peer-to-peer network and a nondestructive way to track data changes over time (Esmaeilian et al. 2020).
- *Autonomous robots*: Autonomous robots are highly intelligent and capable of self-organization, self-evaluation, and decision-making for executing several tasks without human instructions (Bekey, 2005). An autonomous robot can be in various sizes and shapes, and with different levels of autonomy, mobility, and intelligence (Bekey, 2005).
- *Unmanned aerial vehicle (UAV)*: UAV, or commonly referred to as the drone, is a flying device that does not require a human pilot onboard. It is typically piloted by remote control or by a combined control with computer programming (Yang et al. 2020).
- *Additive manufacturing (AM)*: AM, or 3D printing, is a layer-wised production or generative manufacturing. By adding material layer upon layer, it provides opportunities for the accurate production of items at the required size, shape, and material without any wastes (Isasi-Sanchez et al. 2020). With technological maturity and the growing awareness of sustainability, AM has been increasingly used as the main element in both production and logistics processes.
- *Augmented reality (AR)*: AR in the overlaying of computer-generated digital information, e.g., texts, images, and effects, in the real world, which can interact with users and give real-time instructions in a user-friendly way (Anurag 2020)*.*
- *Virtual technologies and simulation:* Virtual technologies are powerful tools, which can mimic, evaluate, optimize, and control a real-world entity or a system in its digital representation under a risk-free and cost-efficient environment.
- *Cybersecurity*: Cybersecurity refers to the protection and defense of critical data, servers and computers, software, and other IT resources from cyber-attacks (Craigen et al. 2014).

## Research method

A systematic literature review aims at identifying, evaluating, interpreting, and categorizing all relevant articles engaging one or more research questions and topics (Kitchenham 2004, Ranieri et al. 2018). Compared with a narrative literature study whose results mainly focus on the descriptive findings of a specific domain of knowledge and may suffer from selection bias, a systematic literature review can present a comprehensive overview of the research landscapes (Evangelista and Durst 2015). Based on Kazemi et al. (2019) and Ren et al. (2020), a systematic literature review consists of the following steps:

1. *Identification of research questions*: Formulating the research questions to be answered.
2. *Literature search and selection*: Developing a document search strategy with a broad combination of keywords to have a comprehensive overview of the area under investigation. Then, proper filters are set up so that the most relevant sample of articles is solicited.
3. *Bibliometric analysis*: Presenting a quantitative analysis and data visualization of the selected sample of articles to understand the key characteristics of the topic, e.g., publication trend, journals and citations, collaborations, and keyword focus.
4. *Content analysis*: Performing a detailed content analysis of the selected articles to summarize the contributions of several related topical areas. Based on this, the current research landscape can be understood, and future research opportunities can be identified.

The research questions are formulated to reflect the aim and scope. This paper links two concepts: sustainable logistics and Industry 4.0, and their interactions in literature are thus focused on. Concerning these concepts, the following three research questions are proposed to understand the state of knowledge of adopting Industry 4.0 technologies in sustainable logistics:

- *RQ1*: What literatures exist on sustainable logistics enabled by Industry 4.0 and how can they be categorized?
- *RQ2*: What are the implications of sustainable logistics in the Industry 4.0 era?
- *RQ3*: What are the future research directions to fill the gaps?

**Fig. 2:**
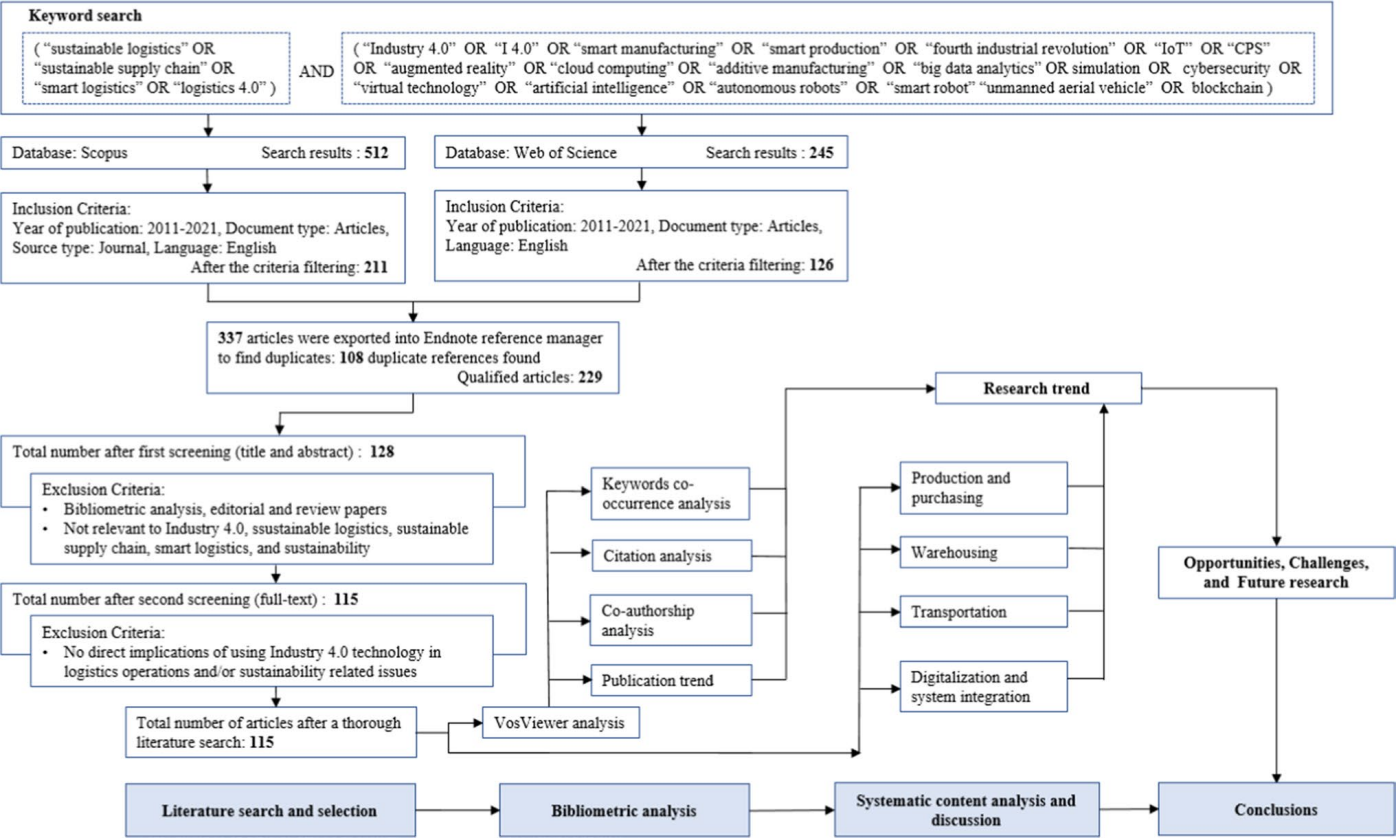
Research method

Based on the research questions above, Fig. 2 formulates the document search strategy, which includes five steps: (1) keyword search, (2) setting of the filters, (3) investigation of the titles and abstracts, (4) investigation of the full text, and (5) result analysis, respectively.

1. *Keyword search*: In this paper, we performed a keyword search using two electronic databases: Scopus and Web of Science core collection. The literature search was conducted in November 2020, and two main sets of keywords related to sustainable logistics and Industry 4.0 were used. The first set of keywords is associated with sustainable and smart logistics, which consist of “sustainable logistics,” “smart logistics,” and “logistics 4.0”. Besides, since many logistics issues were discussed in the context of supply chains, “sustainable supply chain” was added to this group. The other set of keywords related to Industry 4.0 includes “Industry 4.0,” “I4.0,” “smart manufacturing,” “smart production,” “the fourth industrial revolution,” “IoT,” “CPS,” “big data analytics,” “augmented reality,” “cloud computing,” “additive manufacturing,” “autonomous robots,” “smart robot,” “simulation,” “cybersecurity,” “virtual technology,” “artificial intelligence,” “unmanned aerial vehicle,” and “blockchain”. The Boolean operator “OR” was used to combine the keywords within the same group, and “AND” was used to combine the two main groups of keywords related to both sustainable logistics and Industry 4.0. The initial search yielded 512 results in Scopus and 245 in Web of Science.
2. *The setting of the filters:* The second step is to set up several filters to select the most relevant articles, and the papers are excluded if they are not within the research scope or are irrelevant for answering the research questions. First, since the concept of Industry 4.0 was originally presented at the Hannover fair in 2011 (Rojko 2017), the search horizon was re-set to 2011–present. Considering the quality and rigor of selected papers, the search results were also limited to journal articles that had passed the peer-review stage. The publishing language was restricted to English. Thus, conference proceedings, book chapters, pre-prints, and papers published in another language were excluded in this study. After implementing these new filters, the search resulted in 211 and 126 qualified articles in Scopus and Web of Science, respectively. We combined the search results from the two databases and removed the duplicated ones, which resulted in 229 articles.
3. *Investigation of the titles and abstracts*: First, we investigated the type of paper in the filtered sample, 8 bibliometric analysis papers; editorial and review articles were excluded. Then, we investigated the thematic relevance of these articles; papers that have little relevance of using Industry 4.0 and smart technologies in sustainable logistics were excluded. Besides, papers dealing with behavior supply chain issues, e.g., customer relations management, but without a logistics focus, were also excluded. In total, 101 papers were excluded in this stage.
4. *Investigation of the full text*: In the next step, we conducted a full-text reading in the second-round paper selection. In this stage, special emphasis was paid to the papers that lack direct implications for the proposed research questions. Even though these papers have both keywords of Industry 4.0 and logistics or sustainability, the application of Industry 4.0 technologies in sustainable logistics is not thoroughly discussed, so these papers are considered irrelevant to answer the research questions. In this stage, another 13 papers were considered not to fit well with the topic and were thus removed. Then, a total of 115 papers were selected.
5. *Result analysis:* Based on the selected sample, the bibliometric analysis was conducted to provide the results of publication trend, source distribution, co-authorship analysis, citation analysis, and keyword co-occurrence analysis. Next, the content analysis was performed to discuss how different logistics operations can be improved by Industry 4.0 technologies and present the opportunities, challenges, and future research directions.

## Bibliometric analysis

### Publication trend

**Fig. 3:**
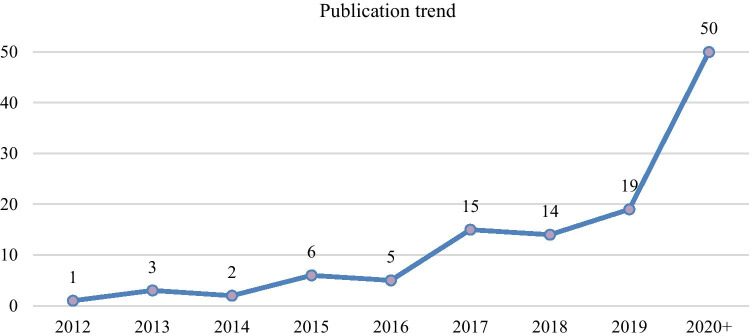
Publication trend of sustainable logistics enabled by Industry 4.0

Figure 3 illustrates the number of articles published between 2012 and 2020. It can be seen that increasing focuses have been given to adopting Industry 4.0 technologies in sustainable logistics planning and operations, and this trend has experienced a significant acceleration since 2017. In 2020 alone, 48 papers have been published in international journals, which amounts to 41.7% of the total publications in the last decade. The publication trend shows that the recent rise of Industry 4.0 related research has presented new opportunities for achieving sustainable value creation, environmental friendliness, and improved social responsibility in logistics activities, which have been noted by both industry professionals and academia.

### Source distribution, influences, and interactions

Table 2 presents the source distribution of the selected 115 articles, which are published in 73 journals. The most popular 15 journals in this field published 57 articles, accounting for nearly 50% of the total amount. With 9 papers published, the *Journal of Cleaner Production* has the highest number of publications, and it is followed by Sustainability with 8 papers. Both are multidisciplinary with the primary focus on theoretical advancements and practices in sustainable development and circular economy. The next three most popular journals are the *International Journal of Production Research*, *Resources Conservation and Recycling*, and *IEEE Access,* contributing to 7, 5, and 5 papers, respectively. Followed by *Industrial Management and Data Systems*, *International Journal of Production Economics*, and *Journal of Self Governance and Management Economics* with 3 articles each. Among the most popular 15 journals, *Sustainability*, *IEEE Access*, and *Applied Sciences* are open access journals, while the others are hybrid journals with both subscriptions only and paid open access options. These 15 journals cover various topics, i.e., sustainable development, production and economics, engineering, computer and data sciences, and logistics and transportation, which shows the cross-disciplinary nature of combining Industry 4.0 and sustainable logistics.

**Table 2:** Source distribution

Publication source (Journal)	Number of papers
*Journal of Cleaner Production*	9
*Sustainability Switzerland*	8
*International Journal of Production Research*	7
*Resources Conservation and Recycling*	5
*IEEE Access*	5
*Industrial Management and Data Systems*	3
*International Journal of Production Economics*	3
*Journal of Self Governance and Management Economics*	3
*Applied Sciences Switzerland*	2
*Chemical Engineering Transactions*	2
*Computers and Electronics in Agriculture*	2
*Economics Management and Financial Markets*	2
*International Journal of Logistics Management*	2
*International Journal of Logistics Research and Applications*	2
*Transportation Research Part E Logistics and Transportation Review*	2
Others (1 per journal)	58

**Fig. 4:**
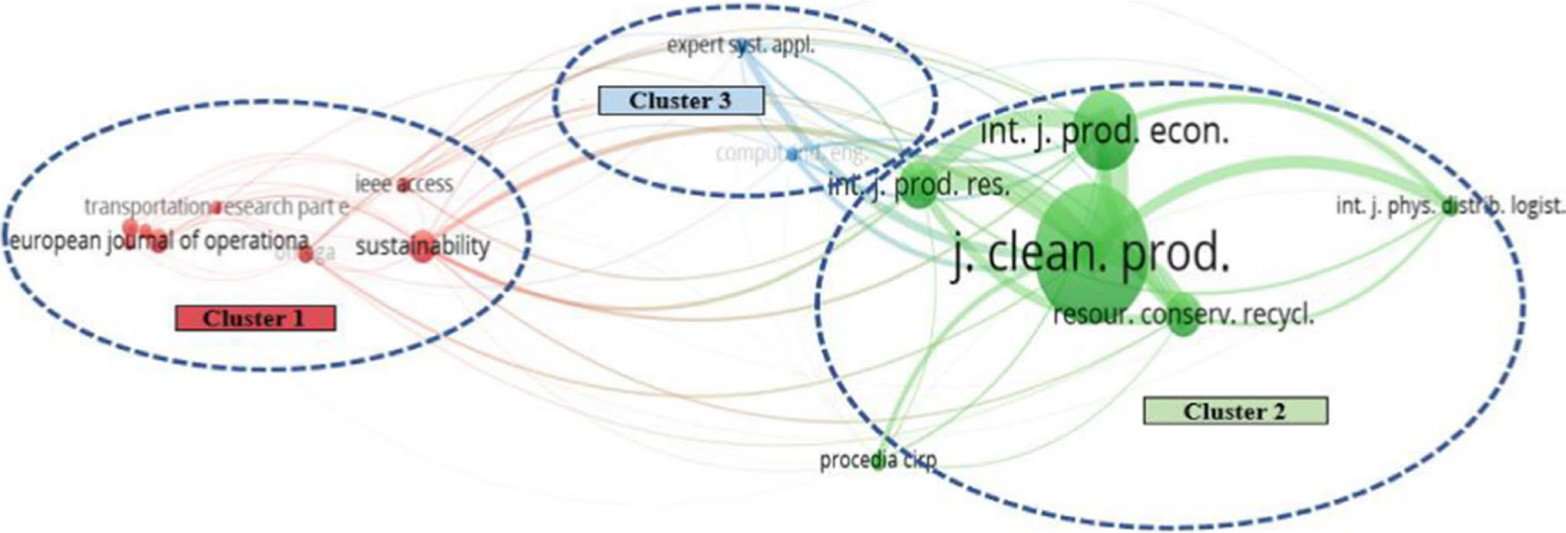
The journal co-citation network

We conducted a co-citation analysis to understand the interactions among the most influential journals in this field. The minimum number of citations per journal was set to 20 in VOSviewer, which led to 16 qualified sources for the co-citation analysis. Compared with the list of journals in Table 2, six new journals were selected namely *Computers & Industrial Engineering*, *Expert Systems and Applications*, *Omega*, *Journal of Operations Management*, *International Journal of Physical Distribution & Logistics Management*, and *Procedia CIRP*. The result is shown in Fig. 4. The size of each node shows the number of citations received by the relevant papers published in each journal, and the arc linking two journals illustrates the co-citation strength between them.

Three general clusters of journals are identified based on their co-citation activities. The first cluster focuses on operations research and operations management. Besides, two inter-disciplinary journals (*Sustainability* and *IEEE Access*) are also assigned to this cluster. The second cluster relates to production technologies and management, while the third cluster emphasizes industrial applications. The journals in the second cluster, particularly the *Journal of Cleaner Production*, *International Journal of Production Economics*, and *International Journal of Production Research*, have yielded the most significant impact and the most active interactions with others in the contemporary research associated with sustainable logistics and Industry 4.0. In addition, the active interactions between clusters 1 and 2, and between clusters 2 and 3 indicate that production-related journals become a bridge to connect the theoretically focused operations research and management methods with real-world industrial applications.

### Influential research, co-authorship network, and co-citation map

Table 3 presents the authors, technologies, applications, and the number of citations of the top ten most influential papers by the time of this research. The most cited article is given by Saberi et al. (2019) in which the relationship between blockchain and sustainable logistics is thoroughly investigated. Followed by Barreto et al. (2017) and Luthra and Mangla (2018), the implications and challenges of Industry 4.0 on logistics activities are discussed. The fourth and fifth highly cited papers are from Prause (2015) and Prause and Atari (2017), which focus on Industry 4.0 enabled architectures of sustainable business models and sustainable manufacturing networks related to logistics operations. In addition, the other papers give comprehensive discussions on the use of several emerging technologies to achieve smart and sustainable logistics, i.e., cloud-enabled product-service system (Zhang et al. 2016), IoT-based smart warehouse management (Lee et al. 2018; Trappey et al. 2017), smart technology–enabled innovative and sustainable business models (Strandhagen et al. 2017), smart decision-making of sustainable logistics (Li et al. 2019), and sustainable logistics practices (Longo, 2012, Hilpert et al. 2013). The results show that, in sustainable logistics systems, the application of several Industry 4.0 technologies, i.e., blockchain, IoT, and cloud-based technologies, has enjoyed tremendous popularity among recent research.

**Table 3:** The top 10 highly cited articles

Papers	Technological keywords	Application focuses	Citations
Saberi et al. (2019)	Blockchain	Sustainable logistics and supply chain	225
Barreto et al. (2017)	Industry 4.0	Logistics operations	132
Luthra and Mangla (2018)	Industry 4.0	Sustainable logistics and supply chain challenges in developing countries	106
Prause (2015)	Industry 4.0	Sustainable business models	60
Prause and Atari (2017)	Industry 4.0	Sustainable production networks and logistics	52
Zhang et al. (2016)	Cloud technology	Product-service oriented cloud logistics	52
Lee et al. (2018)	IoT	Smart warehouse management	47
Strandhagen et al. (2017)	Industry 4.0	Sustainable business innovations for Logistics 4.0	43
Cole et al. (2019)	Blockchain	Logistics and supply chain	40
Li et al. (2019)	Cloud technology	Sustainable logistics and supply chain	34

**Fig. 5:**
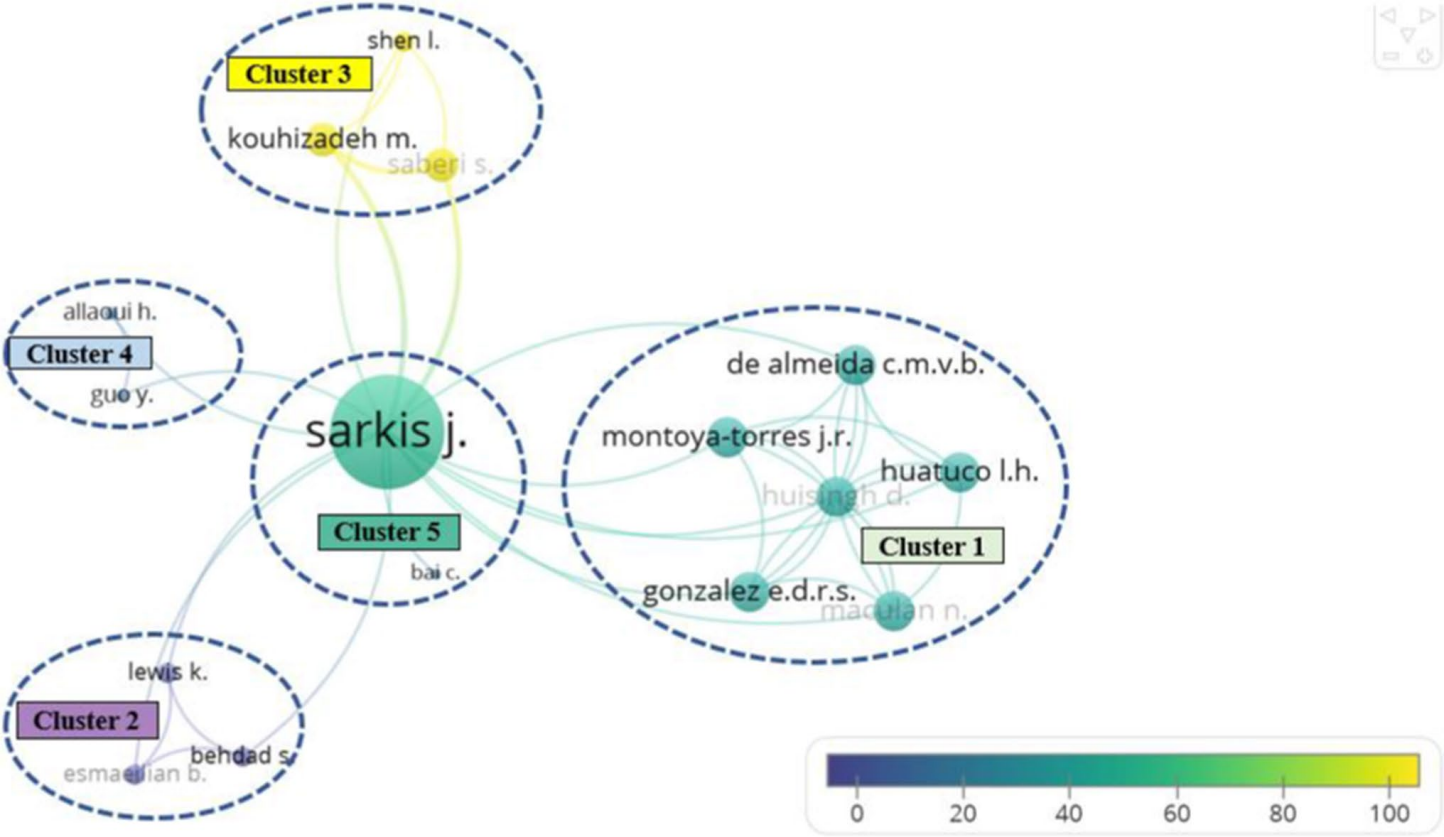
Co-authorship mapping of collaboration

**Fig. 6:**
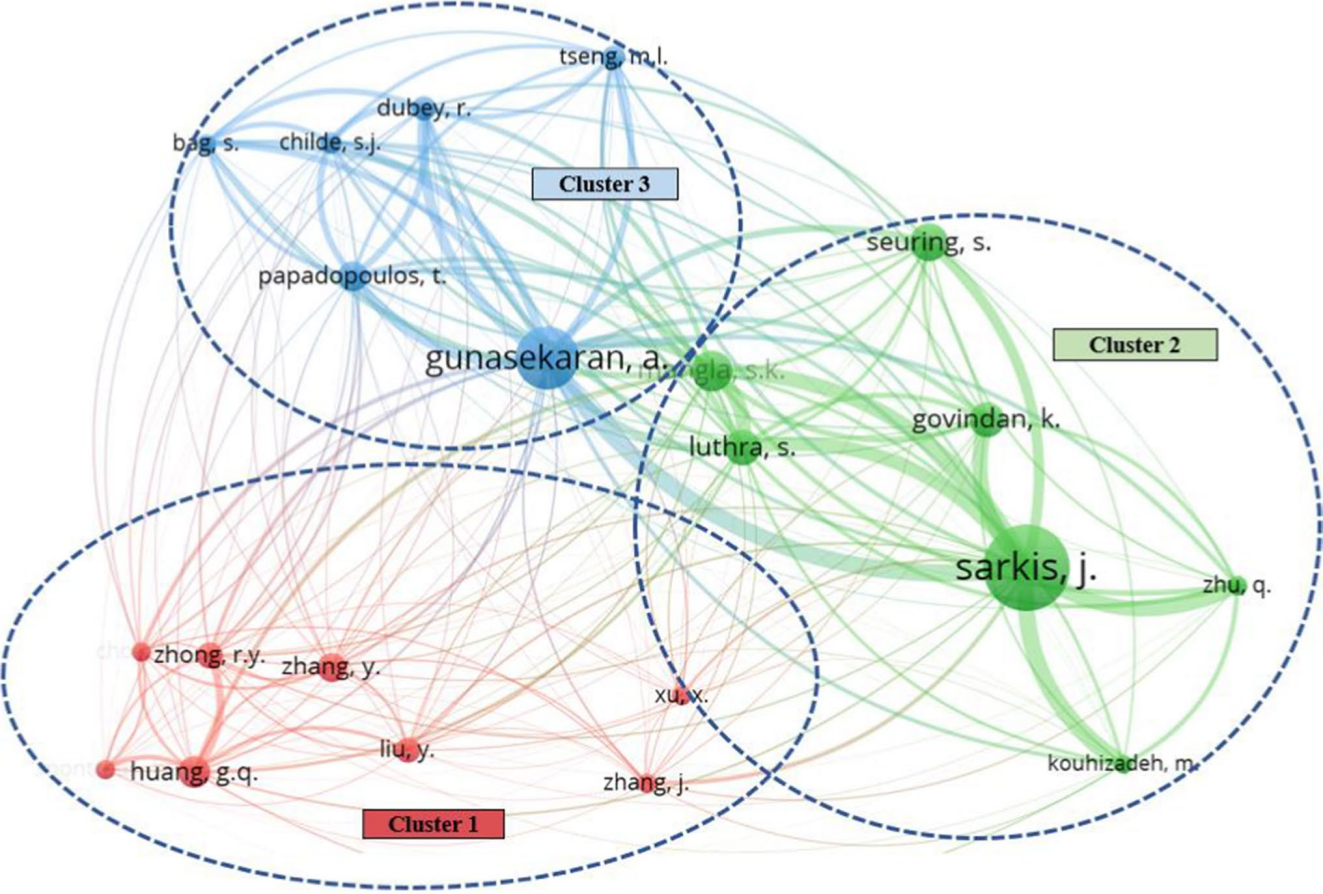
Co-citation map

To identify the most fruitful collaborations and active interactions among different researchers in this field, co-authorship mapping and co-citation mapping are given in Figs. 5 and 6. With the help of VOSviewer, a comprehensive co-authorship network analysis of 363 authors was performed, whose result illustrated the 16 most collaborative authors and their collaborations on the time horizon. The nodes are identified by the authors’ names, whose sizes show the levels of collaborations of different authors. The arcs link these authors with the number of co-authored papers and the time of publications, which are represented by the width and the color of an arc. The total link strength (TLS) of an author is determined by both the number of connecting links and the number of co-authored documents. As shown in Fig. 5, these 16 authors are divided into five clusters with a different number of co-authored papers and citations. The co-citation map in Fig. 6 evaluates the influence of the key researchers and the impacts of their papers on other researchers’ works in sustainable logistics enabled by Industry 4.0. In this analysis, the minimum number of citations per author was set to 20 to identify the most influential researchers who drove the advancement of this field. The results have shown the 21 most influential researchers and their co-citation networks.

Through the comparison between the co-authorship map and the co-citation map, two interesting findings are obtained. First, even though the combination between sustainable logistics and Industry 4.0 has been extensively focused on by worldwide researchers, the collaboration network has not become extensive compared with other well-established fields. This is because this emerging and cross-disciplinary research topic is still at its initial stage. Another reason may be explained by the time from cooperation to publication is usually very long, which may also significantly affect the results of the co-authorship analysis. The second finding is that, even if the collaboration potential has not been fully exploited, several influential researchers and works have led the research and drastically push forward the knowledge accumulation, which forms the foundation to promote fruitful collaboration in the future.

### Research highlights and keywords

**Fig. 7:**
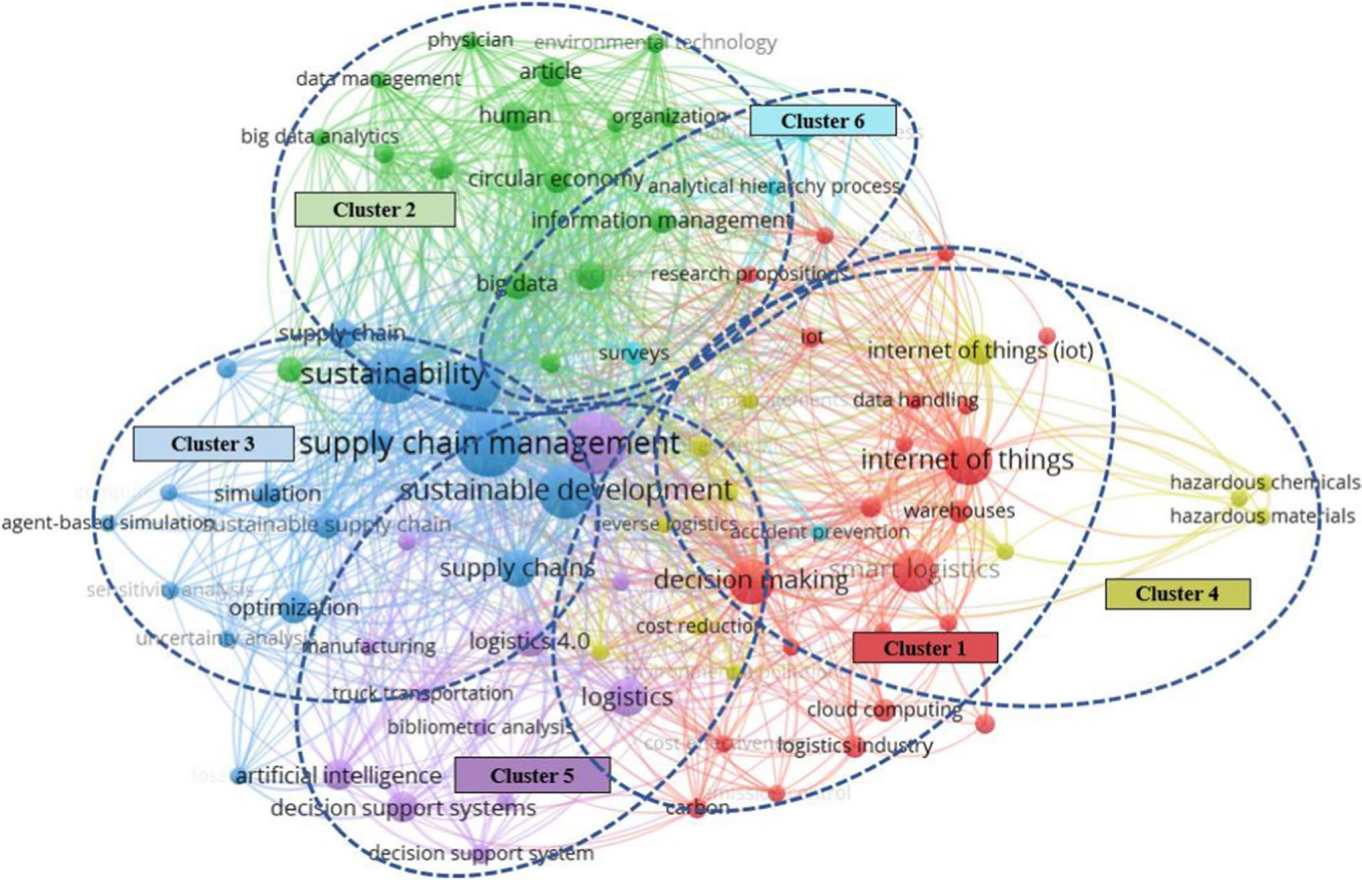
Keyword co-occurrence map

To identify the research highlights, a co-occurrence analysis of the highly used keywords related to Industry 4.0 and sustainable logistics was performed. For presenting a complete overview of the current research landscape, we used “all keywords” and “full counting” options to enumerate all the keywords that appeared in previous studies and calculate the total co-occurrence. With the minimum threshold of three times of co-occurrence, Fig. 7 shows the mapping and interactions of the 78 qualified ones out of the total 1006 keywords. The clusters, occurrences, and TLSs of these keywords are given in Appendix 1 (Table 4).

The 10 mostly used keywords in the selected literatures consist of supply chain management (Occurrence = 39, TSL = 264), Industry 4.0 (Occurrence = 34, TSL = 151), sustainability (Occurrence = 29, TSL = 191), sustainable development (Occurrence = 28, TSL = 220), sustainable supply chains (Occurrence = 28, TSL = 235), internet of things (Occurrence = 21, TSL = 117), decision making (Occurrence = 19, TSL = 115), smart logistics (Occurrence = 17, TSL = 52), logistics (Occurrence = 14, TSL = 59), and supply chains (Occurrence = 13, TSL = 94). Clearly, these mostly used keywords have critical impact and define the general nature of smart and sustainable logistics systems. Besides, it is noted that even if supply chain management and logistics are two concepts, they are not mutually exclusive. Since logistics is considered an important element of supply chain, many relevant studies discuss the sustainable logistics enabled by Industry 4.0 in the context of supply chain management and sustainable supply chain.

The 78 frequently appeared keywords are grouped into six clusters, with which the mainstream research directions on Industry 4.0 enabled sustainable logistics can be pinpointed. The six keyword clusters have identified the different research focuses. Cluster 1 comprises 23 keywords focusing mainly on the application of new technologies, e.g., IoT, cloud computing, etc., in smart warehousing, smart information systems, and other logistics operations. Cluster 2 contains 16 items that predominantly emphasize the use of big data analytics and blockchain to improve sustainable logistics and circular economy. Cluster 3 covers 16 nodes focusing on sustainable logistics operations with optimization and simulation methods. Cluster 4 consists of 11 keywords, which engage in the economic, environmental, and social sustainability of hazardous material management. Cluster 5 includes 8 nodes that focus on improved decision-making with AI and other smart technologies. Cluster 6 consists of 4 keywords related to literature studies, which show efforts have been spent to summarize the recent research results.

## Content analysis

**Fig. 8:**
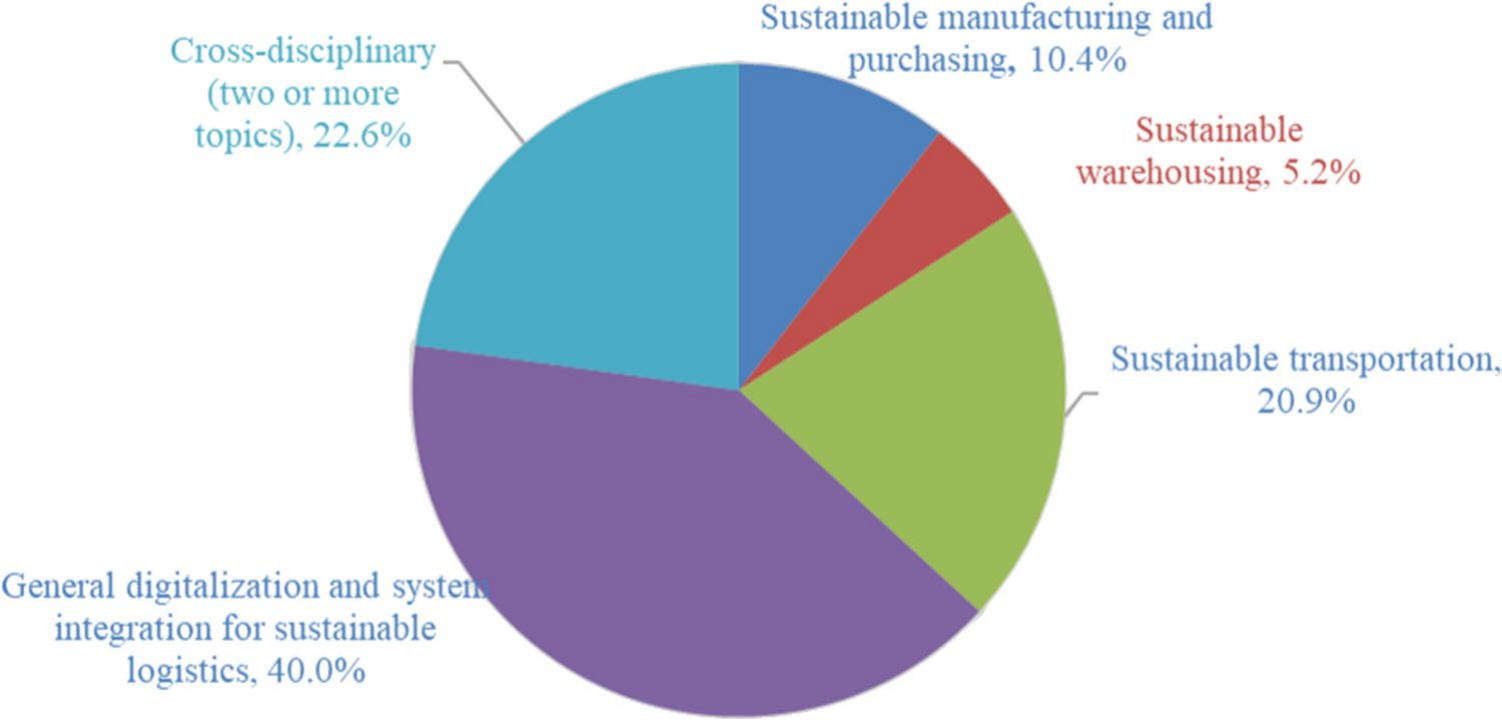
Article distribution over different topical areas

The keyword co-occurrence analysis has shown the importance of technology and data in sustainable logistics, and the content analysis is performed to understand how smart technologies and data analytics will affect the paradigm of logistics operations and the system’s sustainability. Content analysis is an important step to systematically analyze the research development of several topical areas (Kazemi et al. 2019). In this section, we present a detailed content analysis of four main topics related to sustainable logistics operations throughout the pre-production, in-production, and post-production stages. First, smart production drives new demand patterns and changes the way of how demands are satisfied, and it consequently changes the demands of purchasing and logistics services. Thus, Industry 4.0 enabled sustainable production and purchasing was first discussed. The smart solutions for the two most important logistics operations, namely, warehousing and transportation, were then introduced. Last but not least, the general digitalization and system integration issues for streamlining different operations within a sustainable logistics system were given. Figure 8 shows the article distribution over the four topics, and it is noted 22.6% of papers focus on two or more topical areas. A summary of relevant papers, technologies, and sustainability dimensions (environmental or social) of logistics systems in each topic is given in Appendixes 2, 3, 4, and 5 (Tables 5, 6, 7, and 8).

### Industry 4.0 enabled sustainable production and purchasing

In an increasingly globalized and dynamic market, Industry 4.0 technologies play vital roles in improving the sustainability of production operations, purchasing decisions, and resource planning. These technologies can enable efficient commodity flow and information flow from raw material purchasing to product delivery through an open, dynamic, smart, and sustainable production-logistics network (Prause and Atari 2017). Besides, they can affect the capabilities of dynamic remanufacturing, green production, waste reduction, and recycling in a sustainable logistics system (Gonzalez et al. 2015; Björklund and Forslund 2018).

IoT and autonomous robots are the fundamental parts of a smart production system, which allow for a high level of connectivity and automation. IoT-embedded systems can provide better tracking and traceability, which help products move faster and provide customers with real-time information about the deliveries (Bag et al. 2020c). The integration of IoT-enabled devices, autonomous robots, cloud-based data analytics form a connected, digitalized, and smart production CPS. This smart networking of both physical devices and cyber intelligence enables effective machine-to-machine communications and human–machine interactions (Esmaeilian et al. 2020), which pave the way for an autonomous production system with high flexibility and agility.

Big data analysis has gained increasing focus in production and logistics. Advanced analytical tools, i.e., AI and machine learning, have been used to treat a large amount of complex data collected from different sources (Wang et al. 2016). The results can be used to analyze market trends, purchasing patterns, potential risks, equipment maintenance cycles (Samir et al. 2019), delivery reliability and responsiveness (Bag et al. 2020c), and other important performance indicators (Wang et al. 2016), based on which production activities can be planned more sustainably.

AM can be used for on-demand and decentralized production, which allows customers to be actively involved in product design. AM can help to reduce size-related resource constraints (Beltagui et al. 2020), to minimize waste of materials, and to support low-volume and highly customized production, e.g., spare parts (Isasi-Sanchez et al. 2020). The open design architecture of AM facilitates market growth, promotes localized production, generates value-differentiated consumer demands, changes the market leaders’ practices, and supports and diffuses social sustainability in their daily activities (Beltagui et al. 2020).

Cloud technologies provide a platform for centralized storage and decentralized access of various data analytics and computing tools to fulfill the growing demands of mass individualization, improve the responsiveness to customers and market change, and enable broader global cooperation (Strandhagen et al. 2017). The maturity of supplier selection and purchasing strategy can be affected by the effectiveness and timeliness of data exchange with partners (Facchini et al. 2020). In this regard, Ma et al. (2017) presented a sustainable make-to-order apparel supply chain model with a collaborative cloud service platform. The key information, i.e., the order queue of the supplier, the raw material status, and the production capacity can be accessed in real-time, which are used for making sustainable production and purchasing decisions in the apparel industry.

With the high reliability and transparency, blockchain is another highly focused Industry 4.0 technology for the effective integration of information flow and material flow (Saberi et al. 2019). Blockchain can change the way of obtaining, managing, and using the critical product data through the entire product lifecycle, which enables a better product design, more effective production and sales planning, and responsible recovery at the EOL stage (Esmaeilian et al. 2020). From the environmental perspective, blockchain can help to reduce waste and promote recycling. In addition, blockchain traceability can improve social sustainability through a better assurance of human rights, equity, and safety aspects (Saberi et al. 2019). For example, the traceable record of product history allows buyers and producers to trade with high confidence.

### Industry 4.0 enabled sustainable warehousing

Warehouses are the important storage and hub facilities in a logistics network, which provide protection of goods and bridge the gap between different logistics activities, e.g., purchasing and production. Warehouse management consists of four operations, namely, receiving and recording of goods from different suppliers, storing goods at appropriate locations, retrieving and picking goods when they are needed, and shipment to customers (Ten Hompel and Schmidt 2008). Industry 4.0 technologies have brought opportunities for smart and sustainable warehousing solutions with enhanced capability of information and communication-based decision making (Trab et al. 2017). The use of IoT, CPS, AI, and autonomous robots has been investigated in various operations (Lee et al. 2018), e.g., product receiving, identification, storing and allocation (Zhou et al. 2017), and product picking (Rakyta et al. 2016) and shipping with autonomous robots (Trab et al. 2017; Yavas and Ozkan-Ozen 2020).

IoT-enabled devices have been widely used in smart warehouse management by several large companies, i.e., DHL, Amazon, and JD.com. The combination of both IoT and CPS provides a quick interconnection of smart assets in a warehouse, e.g., pallets, forklifts, machines, and robots. This enables real-time data collection and system monitoring of goods, equipment, and personnel, which improves warehousing operations, decision making, safety, and resource utilization (Jabbar et al. 2018; Tang et al. 2020). Besides, by using IoT, cloud technologies, and blockchain (Shoaib et al. 2020), traceability and transparency can be facilitated, and the errors and delays of warehousing operations can be minimized (Lee et al. 2018).

Combining cloud-based data collection, analytics, and optimization enables better communication and positioning of transport vehicles and more accurate prediction of their arrival time in order to optimize the docking slot and achieve just-in-sequence delivery (Barreto et al. 2017; Ding et al. 2020), through which good handing costs, greenhouse gas (GHG) emissions, and truck drivers’ working hours can be reduced. Lv et al. (2020) investigated a data-driven optimization framework for improving the operational efficiency of yard management in steel logistics parks. With the help of smart sensors, AI-supported optimization can adjust the allocations and e-routes of goods and optimize the work assignments with real-time information of available spaces and resources (Munsamy et al. 2020). Besides, these technologies can provide better visibility of inventory levels, enhanced inventory accuracy and space usage (Lee et al. 2018), reduced inventory costs, improved process management and safety (Trab et al. 2017), and better customer services.

Smart robots consist of various sensors and powerful processors that allow them to sense extensively, decide intelligently, and behave precisely (Liu et al. 2018). Smart robots have been increasingly used to replace manual operations, minimize errors, and improve effectiveness and safety. The use of UAVs for picking, data collection, and process monitoring has also been discussed (Gunal, 2019). AM is another emerging technology that has been increasingly used in warehouse management, and it provides an inexpensive solution for holding digital inventory of a large variety of products with low and irregular demands.

Virtual technologies have been extensively adopted to improve the effectiveness and training of warehousing operations. For instance, virtual reality (VR) can be used for the training of new employees without interrupting warehouse operations (Liu et al. 2018), and under minimum risks, it can also be used for providing the training of some dangerous operations, e.g., hazardous materials handling. Simulation has been widely used for visualization, testing, and performance evaluation of new technologies and processes (Azarian et al. 2019). Several logistics companies, e.g., DHL, use AR to manage and control the warehousing processes (Yavas and Ozkan-Ozen 2020), where real-time instructions and task visualizations can be given to the operators in order to provide better assistance and maximize their effectiveness.

### Industry 4.0 enabled sustainable transportation

The transportation of goods among different locations largely determines the sustainability of a logistics system, and Industry 4.0 technologies can be used for improving sustainability in different transportation activities (Sun et al. 2020), e.g., intelligent transportation systems (Wen et al. 2018), vehicle routing, emission reduction (Pan et al. 2020), green-fleet management (Samir et al. 2019), and pick-up and delivery services (Frontoni et al. 2020). The integration of IoT and AI into a cloud-based platform enables real-time data processing and analysis of traffic conditions, vehicle information, dynamic demands, and recourse availability and usage. Combining advanced optimization algorithms, e.g., genetic algorithm and simulated annealing algorithm (Zhang, 2018), the real-time information can be used for better transportation planning and timely decision making to minimize transport delays (Zhao et al. 2020), increase accident responses, reduce fuel consumption and costs, and minimize GHG emissions, noise (Wen et al. 2018) and the population exposure to risks and hazards (Anandhi et al. 2019).

Big data analytics and AI provide computational powers for processing a large amount of multi-sourced data collected from IoT sensors and selecting the right quality and quantity of data for different decision-support tools, and this has led to an increasing focus on data-driven sustainable transportation planning and logistics optimization. Su and Fan (2020) investigated a green vehicle routing system embedded with big data analytics and AI for better transportation of a smart logistics system, where the performances of costs, energy consumption, GHG emissions, and customer services were improved. Data-driven optimization has also been used in the sustainable planning of multimodal transportation (Sun et al. 2018). Through the data-driven capacity balancing and optimization of different transportation modes, the use of low-carbon and environmentally friendly transportation modes has been drastically increased, e.g., a shift from road to rail transport (Dong and Boute 2020), without a significant compromise on cost-effectiveness.

Virtual technologies provide powerful modules to include sustainability in the modeling and analysis of real-world logistics systems (Hoffa-Dabrowska and Grzybowska 2020). Sun et al. (2018) presented a simulation-based analysis for the planning, decision making, and control of a CPS-enabled logistics network. By minimizing the number of trucks with low or empty loads, the simulation improves transportation strategies with reduced fuel consumption, costs, GHG emissions, and truck drivers’ working hours. Simulation models have also been used to show the benefits of resource sharing in sustainable logistics systems (Hoffa-Dabrowska and Grzybowska, 2020). Besides, combined optimization and simulation have been increasingly used in sustainable logistics, e.g., infrastructure design (da Silva et al. 2017) and network optimization (Hong et al. 2019), to take advantage of the strengths of both methods.

Industry 4.0 technologies have changed the ways of goods delivery. The focus on smart and self-driving vehicles, i.e., autonomous trucks and lorries, has shown the potential to reduce the costs, accident rates, and CO2 emissions (Gružauskas et al. 2018). Another game-changing technology is the UAV, which has been used in many countries for the delivery of parcels, foods, medicines, vaccines, and blood samples (Yang et al. 2020). The UAV or combined vehicle-and-drone system becomes appealing for highly agile last-mile delivery services, which has been practiced by several large companies, e.g., Amazon and Walmart (Issaoui et al. 2020). Besides, it also provides a cost-effective solution for the delivery of low-quantity and emergency goods, e.g., medical supplies, to remote areas (Tatham et al. 2017).

Blockchain-based platforms have been used for helping companies track and measure carbon emissions related to their logistics activities (Saberi et al. 2019). Deep learning and AI technologies have shown the value of using digital voice assistance and intelligent information support system in transportation and logistics services, which improve the deliverymen’s working experiences, service levels, and operational efficiency (Hsiao and Chang 2019).

### Digitalization and system integration for sustainable logistics

In general, digitalization is the most important characteristic of an Industry 4.0 enabled logistics system, which aims at the transformation toward fully data-driven operations (Krykavskyy et al. 2019). This digital transformation requires a high-level integration of different smart technologies and systems, which will promote operational excellence and create sustainable value-added opportunities (Ebinger and Omondi 2020). In this regard, many studies have been conducted for enhancing the digitalization and system integration of the entire logistics system.

Many believe IoT-based platforms, which establish the connection between the physical world and the digital world (Tang et al. 2020), are the initial step to achieve a high-level digitalization and system integration of different logistics operations. Big data analytics and AI are digital elements for trend analysis, facility control, risk management, and other logistics operations (Bag et al. 2020b). The cloud-based integration of IoT and AI enables real-time data analytics and optimal decision support. Besides, advanced controlling and autonomous technologies improve the operational efficiency, accuracy, and safety of various logistics activities. Trappey et al. (2017) investigated an IoT- and AI-enabled intelligent logistics system, which improved logistics services by integrating several operations, i.e., machine-loading control, production flow monitoring, vehicle routing, delivery schedules, and vehicle movement tracking.

The multi-sourced real-time information flow not only improves the operations within the border of a company but also paves the way for better resource sharing and demand matching among different companies. Gebresenbet et al. (2018) developed a web-based smart platform for quality control, traceability, and demand matching and optimization of farmers, transporters, and customers in a reverse logistics system for biomass recovery and trading. Liu (2015) investigated a data-driven logistics information system for smart collaborations among different stakeholders, e.g., governments, banks, facilities, service providers, and customers, to achieve rapid decision making, cost reduction, and high-quality services. To evaluate the effectiveness of Industry 4.0 enabled sustainable logistics systems, simulation models can be used to provide quantitative insights. By using simulation models, Zissis et al. (2018) analyzed the cost reduction and service level of the smart collaborations for the home delivery of online groceries.

Considering both economic and environmental sustainability, Mastos et al. (2020) proposed an Industry 4.0 enabled forward-reverse logistics system for effective treatment of hazardous chemicals. At the intra-company level, this system enables effective logistics operations including the data-driven collection of hazardous chemicals, proactive maintenance of equipment, vehicle monitoring, data visualization, and decision optimization. At the inter-company level, a cloud-based collaborative ecosystem is established for effective demand matching and cooperation. From the corporate social sustainability perspective, Daú et al. (2019) discussed the application of IoT and other smart technologies to improve the sustainable practices of healthcare logistics.

Blockchain is another important technology for the digitalization of a logistics system and the integration of smart devices and platforms for data sharing and virtual currency transactions. It improves transparency, traceability, and security at every stage of logistics operations (Kouhizadeh et al. 2021) through the tracking of information, physical components, transactions, and participants’ actions and behaviors (Bai and Sarkis 2020), which facilitates the capability of conflict management (Manupati et al. 2020) and risk mitigation (Kodym et al. 2020) in the entire logistics system. This also paves the way for sustainable collaboration among different stakeholders in a trustworthy business environment (Cole et al. 2019). Besides, the opportunities for using blockchain-based digital systems to improve the environmental performance of logistics operations through life cycle assessment have also been discussed (Zhang et al. 2020).

## Discussions

In this section, the opportunities and challenges for sustainable logistics in the Industry 4.0 era are first discussed, and the suggestions for future research are then given.

### Opportunities

**Fig. 9:**
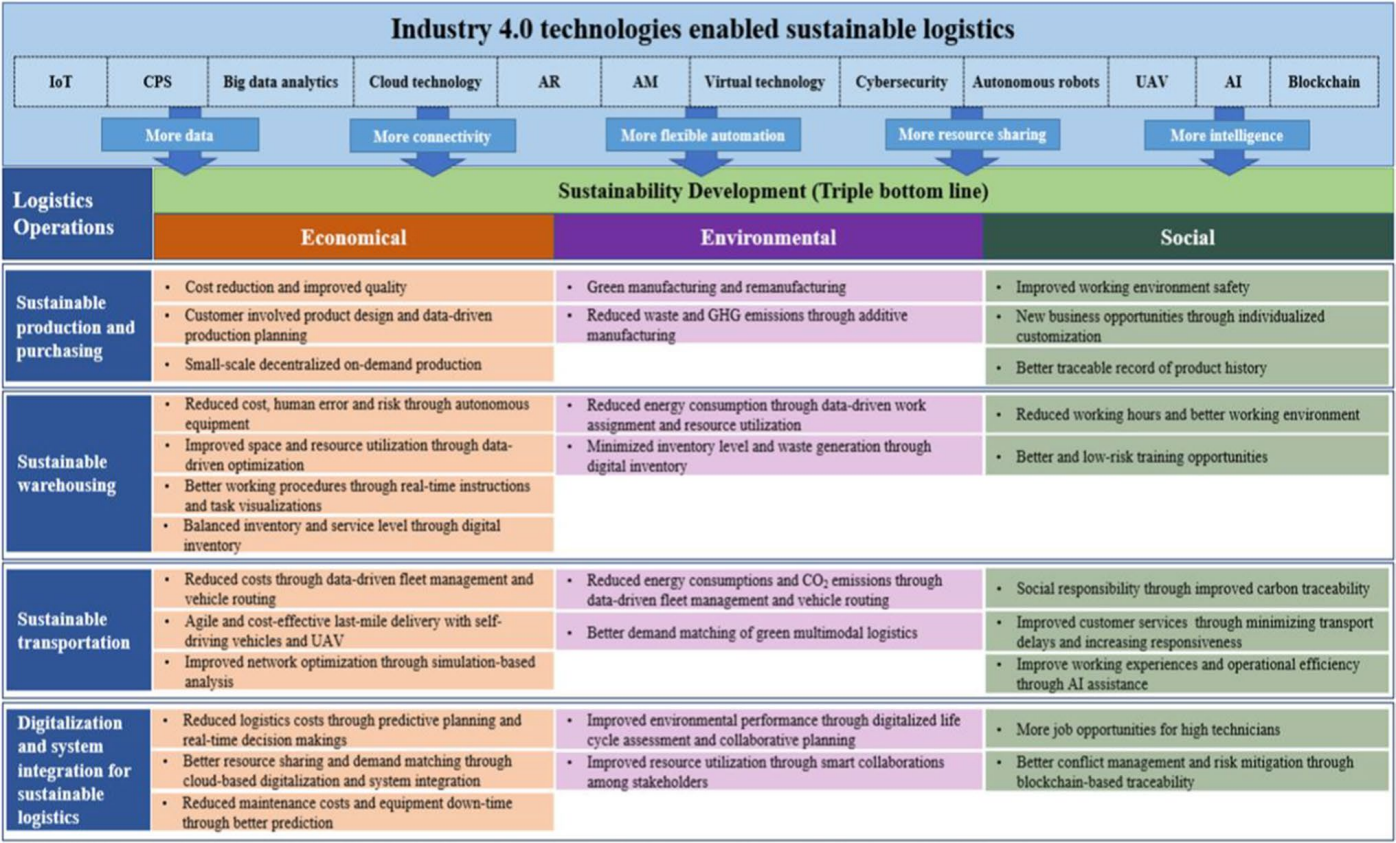
Sustainable logistics enabled by Industry 4.0 technologies

Increasing attention has been paid to improve the sustainability of logistics systems with Industry 4.0 technologies, and worldwide efforts have been spent to advance theoretical development, technology transfer, business model innovation, and industrial applications. Based on the content analysis, Fig. 9 summarizes the impacts of Industry 4.0 technologies on the economic, environmental, and social dimensions of sustainable logistics. The technological revolution provides companies with opportunities to transform their logistics operations to become more responsive to external market changes, while simultaneously being efficient with internal operations. On the one hand, through small-scale localized production with AM and autonomous robots, new business opportunities arise with increasing demands of individualized customizations and product-related services (Yu and Solvang 2017), and this requires service innovation and improvement of logistics operations. Furthermore, the web-based information-sharing systems improve service level and customers’ experiences by a high level of customer involvement throughout the design, production, and delivery processes. On the other hand, the integration of IoT, big data analytics, and AI algorithms via cloud-based platforms provides computing power to handle multi-sourced large volume data, which can be used for better visualization and analysis of some key parameters (Bourke 2019, Abbas and Marwat 2020), i.e., demand trends and maintenance requirements (Li et al. 2017). Furthermore, using better data as inputs to optimization and simulation models, important logistics decisions (da Silva et al. 2017), e.g., production planning, inventory management, routing, and delivery schedules, can be made in a timely and more accurate manner (Zhang 2018).

The most important characteristic of an Industry 4.0 enabled sustainable logistics system is data-driven proactive planning, real-time decision making, and autonomous operations. This high-level digitalization and system integration have led to the conceptual architecture of the digital twin of logistics systems (Ivanov and Dolgui 2020). The digital twin of a logistics system is fully driven by the data collected from both cyber and physical sources (Wang and Wang 2019), e.g., smart sensors and enterprise resource planning (ERP), and it is capable of proactive planning with better analytics of historical data and reactive decision making and scenario analysis with real-time data. From the socio-economic perspective, information sharing among companies in a logistics system and the use of data analytics provide opportunities for better demand matching, resource sharing, and facility usage. The use of autonomous robots and UAV minimizes errors, risks, and labor costs of production, warehousing, and transportation while, simultaneously, provides innovative and environmentally friendly ways of goods delivery (Wen et al. 2018). For instance, logistics information sharing and autonomous equipment are particularly important during the COVID-19 outbreak, which can help to minimize the shortage of emergency medical supplies and to effectively allocate and deliver them to the demand regions.

Better resource planning reduces waste generation and environmental footprints at different stages of a logistics system. Besides, the cloud-based information system provides opportunities to monitor the entire product life cycle and promote effective cloud-based remanufacturing and recycling when they become EOL products (Wang and Wang 2019). From the social sustainability perspective, the adoption of blockchain technologies provides better traceability and more trustworthy business environments in logistics systems. The increased use of autonomous devices improves the safety and working environment of various logistics operations. AI-enabled virtual technologies and AR provide logistics operators with risk-free training, virtual assistance, and real-time task instructions and visualization to improve their working experiences and effectiveness (Ebinger and Omondi 2020, Felstead 2019). In addition, with the requirements of increased digitalization, hardware and software development, and system integration, new job opportunities can be created in the logistics sector as well as in the other related industries.

### Challenges and gaps

Most articles focus on how Industry 4.0 supports sustainable logistics, but less effort has been paid to understand the challenges of this digital transformation. Even though the new technologies have provided many opportunities, they also bring several challenges for sustainable logistics. One example is shown by China’s rapidly developing express delivery and food delivery systems due to the booming of e-commerce. On the one hand, thanks to the better demand allocation and order tracking system empowered by AI, IoT, and advanced optimization, customers can now enjoy cheaper and faster delivery services of their foods and merchandise ordered online. However, on the other hand, these responsive logistics services require much more frequent last-mile deliveries, which lead to more traffic congestions and carbon emissions. Furthermore, the online platform assigns strict requirements to ensure on-time delivery, which raises concerns about the safety issues and job satisfaction of the deliverymen. Thus, we discuss several main challenges and gaps of sustainable logistics in the Industry 4.0 era:

- *Lack of a holistic consideration of multiple sustainable indicators*: A sustainable logistics system balances the trade-off among the economic, environmental, and social dimensions. However, this has not been holistically considered in the adoption of Industry 4.0. For instance, the delivery allocation algorithm of the online food ordering platforms is designed to maximize the service level so that customer satisfaction and economic sustainability can be enhanced, but more environmental and safety issues are not considered holistically.
- *Unclear economic benefits and the impacts of other sustainability indicators:* Compared with the development of technologies, less effort has been spent on the development of quantitative and analytical methods (Karakikes and Nathanail 2017) to evaluate the economic benefits and the impacts of other sustainability indicators by adopting Industry 4.0 in logistics systems. For example, using robots to replace manual operations in a warehouse not only affects a single activity but also largely influences other operations and the performance of the whole logistics system. Several studies show that the lack of concrete evidence on performance improvement has become a major hindrance to confirming companies to adopt Industry 4.0 in their logistics operations (Luthra and Mangla 2018).
- *Lifecycle energy consumption and environmental footprint:* Even if Industry 4.0 shows the potential to reduce waste generation and improve resource utilization, from the lifecycle analysis perspective, the use of a large number of sensors, robots, and other smart devices in various logistics operations has inevitably led to higher energy consumption and potential environmental footprints. For example, UAV is believed an environmentally friendly way to provide responsive and low-carbon delivery service. However, the recent research by Stolaroff et al. (2018) has shown that the environmental footprints of UAV delivery may be higher than the traditional road delivery due to its limited capacity.
- *Job loss and difficulties for workers:* Logistics is a labor-intensive industry, and there is not a high requirement of knowledge and education for first-line operators. On the one hand, the human-centered technological revolution improves logistics operations, efficiency, agility, safety, and working environment. However, on the other hand, from the social sustainability perspective, it will inevitably cause job losses, the anxiety of employees who worry about their careers, and difficulties especially for aging workers both technically and psychologically to adapt to this new transformation.
- *Inequity issues:* The paradigm change of sustainable logistics is led by the market leaders like DHL, Amazon, and JD.COM, who spent large investments for developing smart and autonomous logistics solutions (Olsen and Tomlin 2020). However, the lack of financial and technological resources of small and medium-sized enterprises (SMEs) significantly hinders the adoption of smart technologies and sustainable practices in their logistics systems, which results in an unequal position in the competition with large companies. In addition, studies also reveal that some Industry 4.0 technologies, e.g., big data analytics, can be used for unfair price discrimination and dynamic pricing throughout different players in a logistics system (Jagtap et al. 2021).
- *Lack of a general guideline****:*** The current research focuses on the adoption of an individual or several Industry 4.0 technologies in sustainable logistics. However, the practices are ad-hoc endeavors, and there is a lack of systematic guidelines to link different Industry 4.0 technologies and sustainable logistics operations at various stages.
- *System integration and interoperability:* The meaning of interoperability is that different smart devices and systems can independently communicate and access each other’s functions (Phuyal et al. 2020). Implementing Industry 4.0 in a sustainable logistics system that has many devices for different operations requires not only technological upgrades but also system integration with existing equipment. The communication protocols and control methods of the existing equipment and the new devices or system are by no means identical, so large efforts and upfront costs are required to make the existing logistics system more autonomous, smart, and sustainable.
- *Data quality and cybersecurity concerns: *Industry 4.0 requires effective data sharing horizontally among different facilities and companies in a logistics system and vertically throughout different functions and operations within a company (Foidl and Felderer 2015). However, the maturity and quality of data processing at different companies may not be at the same level, so this technological transformation requires collaborative efforts from various stakeholders in a logistics network. Besides, the concerns of cybersecurity and data safety also hinder the adoption of Industry 4.0 in logistics operations (Phuyal et al. 2020).

In addition, there exist knowledge siloing of current research. Industry 4.0 enabled sustainable logistics is a topic related to several subjects, e.g., computer and data sciences, automation and control, robotics, operations research, and social science. However, from the bibliometric analysis, it is evident that there is still a lack of effective research cooperation among researchers and groups with different backgrounds and geographical locations. This limits the generation of a general guideline and solution that can be widely applicable in different regions.

### Future research suggestions

Several research directions are recommended to fill the literature gaps and better support this smart transformation of sustainable logistics:

1. *Human-centric smart logistics transformation *needs to be focused on. The latest concept of Industry 5.0 extends the technology-centric transformation of Industry 4.0 to a more socially sustainable human-centric transformation, and future research is thus needed to understand how human-centric smart transformation can be achieved in logistics sectors. Besides, several social impacts such as the demographic change and the impacts to aging workers for adopting these smart technologies in logistics sectors need to be better understood.
2. *Multi-objective balanced system design* for sustainable logistics operations. This requires new algorithms and systems that are designed to help with decision-making considering multiple objectives. For example, in the order allocation algorithm for food delivery assignment, a balance needs to be achieved among the service level, the environmental footprints, and the deliverymen’s safety issues in the real-time traffic condition.
3. *The lifecycle environmental impact* of Industry 4.0 enabled logistics systems needs to be better analyzed. Future studies are needed to provide deeper insights into the environmental footprints through lifecycle analysis. For instance, the system boundary of the analysis should be extended to the energy consumption and resource usage related to the production and recycling of the smart devices used in sustainable logistics.
4. *Analytical models and optimization *for adopting Industry 4.0 technologies in smart ways in different logistics operations and for providing quantitative implications of the cost benefits, different sustainability indicators, and overall system performance. Besides, in the Industry 4.0 era, the logistics system’s effectiveness, efficiency, flexibility, agility, and environmental footprints need probably to be re-balanced (Olsen and Tomlin 2020), which requires better-designed analytical tools and optimization algorithms.
5. *The digital twin* of sustainable logistics systems needs to be focused to provide end-to-end solutions to various logistics operations. Via a cloud-based system, the predictive analytics with AI and the real-time data from IoT sensors as well as other cyber and physical portals need to be seamlessly connected with analytical optimization and simulation tools to improve both proactive and real-time decision making in sustainable logistics operations. Besides, future research is also suggested to develop the bi-directional control architecture to achieve highly autonomous logistics operations, e.g., warehousing.
6. *Semi-autonomous sustainable transportation solutions:* Even if autonomous driving vehicles have been extensively focused on in recent years, the realization of a fully autonomous transportation system faces many challenges and uncertainties, i.e., legal restrictions, technological maturity, and safety issues. Thus, the use of semi-autonomous solutions is an attractive alternative for sustainable logistics solutions. For example, truck platooning is a semi-autonomous system that has several benefits, e.g., improved efficiency, reduced labor costs, and reduced fuel consumption and carbon emissions due to the reduction of the aerodynamic resistance of the following trucks.
7. *Broad and diversified technology focus* should be given not only to IoT, CPS, AI, and autonomous robots but also to others, e.g., AR and AM, which receive much less attention in the literature related to sustainable logistics. Thus, future research is needed to provide a better understanding of how those Industry 4.0 can be used to enhance sustainable logistics operations.
8. *Sustainable reverse logistics* enabled by Industry 4.0 is another opportunity for future research. Less focus has been given to the smart transformation of reverse logistics, which faces challenges related to the uncertainty of the market demands and the quantity and quality of the returned products. Smart technologies provide opportunities for minimizing the impact of uncertainty with better prediction and for more effective resource sharing among different companies. Besides, the use of AI-enabled autonomous robots has shown potentials to replace human workers from harsh working environments, e.g., manual sorting of waste. In addition, the terms “demand individualization” and “individualized customization” need to be redefined in reverse logistics.
9. *The smart and sustainable logistics solutions for the COVID-19 *need to be focused on. Due to the rapidly increased demand, strict border control and city lockdown, and reduced transportation capacity, the COVID-19 pandemic has hindered the flows of goods, increased logistics costs, and imposed a higher risk on vulnerable groups related to the shortage of medical supplies, foods, and other necessities. In this regard, the role of Industry 4.0 technologies needs to be highlighted to tackle the logistics challenges during the pandemic. For example, the use of robots to collect infectious waste at healthcare facilities may reduce the infection risks.

## Conclusions

The concepts of sustainable logistics and Industry 4.0 have been focused on by many researchers due to the increasing need for technology-driven smart and sustainable logistics. This paper provides a systematic literature review focusing on the recent development and adoption of various Industry 4.0 technologies in sustainable logistics at both intra- and inter-company levels. First, a bibliometric analysis was conducted to identify the publication trend, the most influential journals and research, the co-citation networks, and the most frequently used keywords. Then, a content analysis was performed to understand the current research landscape on how Industry 4.0 technologies can be used to improve sustainable logistics activities, namely, production and purchasing, warehousing, transportation, and general system integration. Finally, current research developments were summarized, and the challenges, literature gaps, and future research opportunities were discussed. To answer the proposed research questions:

- *RQ1*, we systematically analyze the state-of-the-knowledge of Industry 4.0 and sustainable logistics with both bibliometric analysis and content analysis in the “Bibliometric analysis” and “Content analysis” sections.
- *RQ2*, we thoroughly discuss both the opportunities and the challenges of sustainable logistics in the Industry 4.0 era in the “Opportunities” and “Challenges and gaps” sections.
- *RQ3*, we identify 9 future research directions in the “Future research suggestions” section.

From the research perspective, the results show an increasing focus has been given to Industry 4.0 enabled sustainable logistics, which is an area attracting worldwide researchers and practitioners. The results suggest that cooperation among researchers should be enhanced in the future. In addition, the paper analyses the current research landscape and how the paradigm shift of various logistics activities is driven by technological advancements, and their implications on improving economic, environmental, and social sustainability are discussed. Based on this, the gaps and opportunities are identified to guide future research.

From the practical perspective, the results provide insights for the potential application of Industry 4.0 technologies to enhance sustainable logistics practices, which will promote knowledge transfer from academic research to industrial applications. In particular, the use of data-driven decision-support platforms may provide cost-effective, safe, and reliable tools for a better understanding and analysis of the effectiveness and challenges of adopting new technologies, digital solutions, and business models in sustainable logistics systems. From a broader perspective, these discussions also provide other companies with implications and some successful examples for guiding their logistics transformations in the Industry 4.0 era. Besides, the challenges of Industry 4.0 to sustainable logistics systems need to be noticed in this technological and operational transformation. One should bear in mind that the benefits should never be overestimated, and the challenges and commitments required should never be underestimated.

The paper inevitably has several limitations regarding the filters used in the sample selection, which only account for the journal articles published in English with respect to the selected keywords, the two databases, and the time of the search. Sustainable logistics and Industry 4.0 are extensively focused by worldwide researchers, and some important publications may be published in different languages. Besides, both concepts are currently getting fast-growing attention, and relevant papers may be published in different forms, i.e., conference papers, book chapters, magazines, industrial reports, or under the peer-review stage as pre-prints. Therefore, the results presented in this paper are not exhaustive, and future improvements are needed to present a more comprehensive analysis with an extended sample selection.

## Data Availability

Not applicable.

## Appendix 1

**Table 4:** The clusters, the occurrences, and the TLSs of the highly used keywords (The maximum number of keywords included from each cluster is 10)

Clusters	Keywords	Occurrences	TLS
Cluster 1	Internet of things	21	117
	Decision making	19	115
	Smart logistics	17	52
	Cloud computing	5	18
	Logistics industry	5	18
	Warehouses	5	21
	Carbon	4	24
	Embedded systems	4	29
	Information platform	4	8
	IoT	4	21
Cluster 2	Article	8	100
	Big data	8	61
	Blockchain	8	75
	Human	8	100
	Circular economy	7	68
	Information management	6	50
	Sustainable supply chain management	6	36
	Economic aspect	5	58
	Data analytics	4	50
	Environmental sustainability	4	46
Cluster 3	Supply chain management	39	264
	Sustainability	29	191
	Sustainable development	28	220
	Sustainable supply chains	28	235
	Supply chains	13	94
	Optimization	10	51
	Supply chain	8	56
	Simulation	7	32
	Sustainable supply chain	7	46
	Environmental impact	5	43
Cluster 4	Cost reduction	3	16
	Design/methodology/approach	4	32
	Economic and social effects	3	28
	Environmental pollutions	3	20
	Hazardous chemicals	3	13
	Hazardous materials	3	13
	Hazards	3	13
	Industrial economics	4	35
	Industrial research	3	21
	Internet of things (iot)	9	68
Cluster 5	Industry 4.0	34	151
	Logistics	14	59
	Logistics 4.0	10	18
	Artificial intelligence	9	53
	Decision support systems	9	57
	Decision support system	4	29
	Bibliometric analysis	3	16
	Food supply	3	17
	Industrial revolutions	3	14
	Manufacturing	3	14
Cluster 6	Surveys	5	37
	Analytic hierarchy process	3	27
	Analytical hierarchy process	3	27
	Analytic hierarchy process (ahp)	3	21
	Accident prevention	3	16

## Appendix 2

**Table 5:** Industry 4.0 enabled sustainable manufacturing and purchasing

Author/year	Industry 4.0 technology												Sustainable logistics dimension	
	IoT	CPS	Big data	Cloud tech	AR	AM	Virtual tech	Cybersecurity	Autonomous robots	UAV	AI	Blockchain	Environmental	Social
Strandhagen et al. (2017)	√	√	√	√	√	√			√		√		√	√
Barreto et al. (2017)	√	√	√	√									√	√
Facchini et al. (2020)	√	√	√	√	√				√					
Chong et al. (2018)	√	√												
Sutawijaya and Nawangsari (2020)	√		√						√		√		√	
Motevalli-Taher et al. (2020)							√						√	√
Saberi et al. (2019)												√	√	√
Bag et al. (2020c)	√		√								√		√	√
Beltagui et al. (2020)						√								√
Esmaeilian et al. (2020)	√	√		√	√	√		√	√		√	√	√	√
Prause and Atari (2017)		√												
Samir et al. (2019)	√		√	√		√						√		
Isasi-Sanchez et al. (2020)		√				√							√	√
Wang et al. (2016)													√	√
Björklund and Forslund (2018)													√	√
Tuffnell et al. (2019)	√	√	√	√	√								√	
Sheares (2020)	√	√	√		√	√			√		√	√		√
Nica (2019)	√	√									√			√
Felstead (2019)	√	√									√			
Li et al. (2019)													√	√
Bourke (2019)	√										√			√
Gonzalez et al. (2015)													√	√
Ma et al. (2017)							√							

## Appendix 3

**Table 6:** Industry4.0 enabled sustainable warehousing

Author/year	Industry 4.0 technology												Sustainable logistics dimension	
	IoT	CPS	Big data	Cloud tech	AR	AM	Virtual tech	Cybersecurity	Autonomous robots	UAV	AI	Blockchain	Environmental	Social
Barreto et al. (2017)	√	√	√	√									√	√
Ding et al. (2020)	√		√	√							√			
Abbas and Marwat (2020)	√	√					√							
Yavas and Ozkan-Ozen (2020)	√	√			√								√	
Munsamy et al. (2020)	√	√	√	√	√	√	√	√	√	√			√	
Issaoui et al. (2020)	√	√	√	√							√	√	√	√
Lv et al. (2020)	√		√											√
Shoaib et al. (2020)	√											√		√
Rakyta et al. (2016)			√										√	√
Sciortino et al. (2016)			√										√	
Wen et al. (2018)	√		√						√	√	√		√	√
Trab et al. (2017)	√													
Jabbar et al. (2018)	√													
Lee et al. (2018)	√	√												
Samir et al. (2019)	√		√	√		√						√		
Wang et al. (2016)													√	√
Zhou et al. (2017)														
Cui (2017)	√								√					
Tang et al. (2020)							√						√	√

## Appendix 4

**Table 7:** Industry 4.0 enabled sustainable transportation

Author/year	Industry 4.0 technology												Sustainable logistics dimension	
	IoT	CPS	Big data	Cloud tech	AR	AM	Virtual tech	Cybersecurity	Autonomous robots	UAV	AI	Blockchain	Environmental	Social
Barreto et al. (2017)	√	√	√	√									√	√
Facchini et al. (2020)	√	√	√	√	√				√					
Ding et al. (2020)	√		√	√							√			
Liu (2015)			√											√
Yavas and Ozkan-Ozen (2020)	√	√											√	
Munsamy et al. (2020)	√	√	√	√	√	√	√	√	√	√			√	
Issaoui et al. (2020)	√	√	√	√							√	√	√	√
Sutawijaya and Nawangsari (2020)	√		√						√		√		√	
Su and Fan (2020)			√								√		√	√
Mehmann and Teuteberg (2016)							√						√	√
Saberi et al. (2019)												√	√	√
Pan et al. (2020)													√	√
Zhao et al. (2020)	√												√	
Greif et al. (2020)	√						√							
Yang et al. (2020)									√	√	√		√	√
Frontoni et al. (2020)														
Hoffa-Dabrowska and Grzybowska (2020)													√	
Dong and Boute (2020)													√	
Esmaeilian et al. (2020)	√	√		√	√	√		√	√		√	√	√	√
da Silva et al. (2017)							√						√	
Hilpert et al. (2013)													√	
Sciortino et al. (2016)			√										√	
Sivamani et al. (2014)	√													√
Zhang (2018)	√	√	√	√										
Wen et al. (2018)	√		√						√	√	√		√	√
Teucke et al. (2018)	√						√							
Hong et al. (2019)							√						√	√
Sundarakani et al. (2019)														√
Luo and Fu (2017)													√	√
Anandhi et al. (2019)	√													√
Hsiao and Chang (2019)											√			√
Samir et al. (2019)	√		√	√		√						√		
Wang et al. (2016)													√	√
Yu et al. (2015)														
Björklund and Forslund (2018)													√	√
Cho and Kim (2017)	√													√
Benotmane et al. (2017)				√										
Tatham et al. (2017)										√				√
Gružauskas et al. (2018)	√	√	√	√			√						√	
Sun et al. (2018)							√						√	√
Lin et al. (2019)	√						√							
Wanke et al. (2015)							√						√	
Tang et al. (2020)							√						√	√

## Appendix 5

**Table 8:** Industry 4.0 enabled digitalization and system integration for sustainable logistics

Author/year	Industry 4.0 technology												Sustainable logistics dimension	
	IoT	CPS	Big data	Cloud tech	AR	AM	Virtual tech	Cybersecurity	Autonomous robots	UAV	AI	Blockchain	Environmental	Social
Strandhagen et al. (2017)	√	√	√	√	√	√			√		√		√	√
Barreto et al. (2017)	√	√	√	√									√	√
Kucukaltan et al. (2020)			√	√		√			√	√				√
Abbas and Marwat (2020)	√	√					√							
Liu (2015)			√											√
Luthra and Mangla (2018)	√	√	√										√	√
Yavas and Ozkan-Ozen (2020)	√	√											√	
Allaoui et al. (2019)													√	√
Tseng et al. (2019a)			√										√	√
Kumar et al. (2021)	√	√		√		√							√	√
Sutawijaya and Nawangsari (2020)	√		√						√		√		√	
Motevalli-Taher et al. (2020)							√						√	√
Cole et al. (2019)												√		√
Zhang et al. (2016)	√	√		√									√	
Tang (2020)	√		√	√										
Greif et al. (2020)	√						√							
Correa et al. (2020)	√		√											√
Kodym et al. (2020)	√	√	√	√		√		√				√	√	√
Bag et al. (2020c)	√		√								√		√	√
Cui et al. (2020)	√													
Ebinger and Omondi (2020)	√		√	√							√	√	√	√
Mastos et al. (2020)	√	√	√		√								√	
Chiappetta Jabbour et al. (2020)			√										√	√
Morella et al. (2020)	√	√	√	√	√	√	√	√	√				√	
Bag et al. (2020b)			√											√
Yadav and Singh (2020)												√		√
Esmaeilian et al. (2020)	√	√		√	√	√		√	√		√	√	√	√
Yadav et al. (2020)													√	√
Manupati et al. (2020)												√	√	
Shoaib et al. (2020)	√											√		√
Bai and Sarkis (2020)												√		√
Rejeb and Rejeb (2020)												√	√	√
Rakyta et al. (2016)			√										√	√
Daú et al. (2019)	√												√	√
Krykavskyy et al. (2019)														√
Hasan et al. (2020)														
Xie (2018)	√													√
Trappey et al. (2017)	√		√	√										
Anandhi et al. (2019)	√													√
Gebresenbet et al. (2018)														
Bosona et al. (2018)														
Liu et al. (2020b)	√		√										√	
Bag et al. (2020a)	√	√	√	**√**										
Liu et al. (2020a)			√										√	√
Lee et al. (2016)													√	
Sahay and Ierapetritou (2013)							√							
Byun et al. (2014)	√													√
La Scalia et al. (2017)							√						√	
Zhang et al. (2020)	√		√									√	√	√
Ma et al. (2020)													√	
Bricher and Müller (2020)											√			√
Cimini et al. (2019)	√	√	√	√										√
Prause (2015)	√	√	√			√							√	√
Dai (2018)	√		√											√
Dominguez et al. (2020)							√						√	
Gružauskas et al. (2018)	√	√	√	√			√						√	
Tseng et al. (2019b)													√	
Xu et al. (2013)	√		√											
Bukowski (2019)	√		√	√										
Ma et al. (2017)							√							
Zissis et al. (2018)							√						√	√
